# Postdictive Modulation of Visual Orientation

**DOI:** 10.1371/journal.pone.0032608

**Published:** 2012-02-29

**Authors:** Takahiro Kawabe

**Affiliations:** Human and Information Science Laboratory, NTT Communication Science Laboratories, Atsugi, Kanagawa, Japan; Rutgers University, United States of America

## Abstract

The present study investigated how visual orientation is modulated by subsequent orientation inputs. Observers were presented a near-vertical Gabor patch as a target, followed by a left- or right-tilted second Gabor patch as a distracter in the spatial vicinity of the target. The task of the observers was to judge whether the target was right- or left-tilted (Experiment 1) or whether the target was vertical or not (Supplementary experiment). The judgment was biased toward the orientation of the distracter (the postdictive modulation of visual orientation). The judgment bias peaked when the target and distracter were temporally separated by 100 ms, indicating a specific temporal mechanism for this phenomenon. However, when the visibility of the distracter was reduced via backward masking, the judgment bias disappeared. On the other hand, the low-visibility distracter could still cause a simultaneous orientation contrast, indicating that the distracter orientation is still processed in the visual system (Experiment 2). Our results suggest that the postdictive modulation of visual orientation stems from spatiotemporal integration of visual orientation on the basis of a slow feature matching process.

## Introduction

The judgment of momentary visual appearance of a visual object is strongly affected by the temporal context in which the judgment is made. For example, the location of a visual object is judged with a bias in the direction of leading motion [1], [2] or trailing motion [3], [4]. The temporal context dependence of object mislocalization indicates that the visual system determines the momentary visual appearance on the basis of spatiotemporally sluggish processing. The present study focuses on the postdictive modulation of visual appearance by subsequent sensory inputs.

Previous studies have suggested that not only visual location but also other visual features are subject to postdictive modulation. For example, in a two-frame apparent motion, the shape of a visual flash in the initial frame is perceived to gradually transform into the shape of a flash in the second frame on a motion trajectory (plastic deformation [5]–[7]). Plastic deformation is considered to be a kind of postdictive modulation of visual signals because the gradual change of a flash shape should be produced after the flash in the second frame is presented. Interestingly, the color of a flash in the first frame is not perceived to gradually change to the color of a flash in the second frame [8], which is consistent with the fact that the color is not integrated in non-smooth apparent motion [9]. Other studies have shown that the perceived size of a flash is modulated by the physical size of a flash in subsequent frames [10], [11]. This postdictive modulation of visual size perception occurs in an object-based manner [11].

Recent literature suggests that this kind of postdictive modulation of visual appearance is related to object updating. To create a spatiotemporally continuous perceptual world, the visual system has to determine whether a sensory signal in the present moment comes from an object that has already been represented in the brain. If the signal is judged to stem from a previously registered object, the earlier representation of the object is suppressed and consequently updated to the new one. This makes it hard for observers to report the old appearance of the object when object-updating occurs, as demonstrated in studies on object-substitution masking [12]–[15], backward masking [16], and visible persistence along a motion trajectory [17]. A previous study [15] suggested that the plastic deformation in apparent motion as described above is related to object updating, and a close relationship between object-updating and visual motion has been confirmed in a transcranial magnetic stimulation study [18].

During the updating of a visual representation within an object, the old representation of the object is integrated with the new one. The integration produces two perceptual outcomes: The first one is the suppression of the old representation. This has been confirmed by previous studies on motion deblurring. Motion deblurring, a perceptual phenomenon in which the visible persistence of a moving object is suppressed on its motion path [19], is related to signal summation by means of spatiotemporally elongated receptive fields [20], [21]. Interestingly, Moore et al. (2007) [17] demonstrated that motion deblurring occurs only when object updating is maintained along a motion trajectory. Thus, the suppression of the old representation is driven only when the object continuity is spatiotemporally maintained. The second outcome of the integration is bias in the visual appearance of the old representation towards the new one. It has been shown that the change in appearance of a previously viewed target is induced by subsequent nearby maskers and that this influences the determination of apparent motion direction [22]. Similarly, it has also been demonstrated that the flash size in the first frame is reported with a bias toward the flash size in the second frame in an apparent motion display [11].

In this way, previous studies have shown a close relationship between the postdictive modulation of visual appearance and object updating. However, the investigations have been limited to the postdictive modulation of the shape [5]–[7], size [10], [11], and location [23], [24] of objects. In addition, although previous studies have specified that the postdictive modulation of visual location grounds on the spatiotemporal integration of visual signals during a period of 80–100 ms [3], [10], [23], [24], the temporal course of the postdictive modulation of other types of visual signals is still not fully understood.

The present study investigated the postdictive modulation of visual orientation. We had the two specific purposes. The first was to explore whether the judgment of visual orientation could be postdictively modulated. We focus on visual orientation as a subject of investigation for three reasons. First, visual orientation is one of the elementary visual features, and it is unclear whether such an elementary visual feature is involved in the postdictive modulation. Second, it is easy to control the magnitude of tilt as many previous studies have done so. Third, no previous studies have examined the postdictive modulation of visual orientation. The second purpose was to explore the influence of the visibility of subsequent inputs on the postdictive modulation of visual orientation. How postdictive modulation occurs depends on motion correspondence [11], and the motion correspondence is occasionally determined based on higher-order visual feature matching [25]. Thus, the postdictive modulation of visual orientation should vanish when backward masking lowers the visibility of the subsequent orientation, if the postdictive modulation depends on the temporal integration of visual signals on the basis of higher-order feature matching process. On the other hand, some previous studies have demonstrated that the visual orientation is processed even without visual awareness in the visual system [26]–[30]. Thus, the invisible orientation information would contribute to the feature matching, resulting in the postdictive modulation of visual orientation. On the basis of the results, we discuss a putative perceptual mechanism underlying the postdictive modulation of visual orientation.

## Results

### Experiment 1

**Figure 1:**
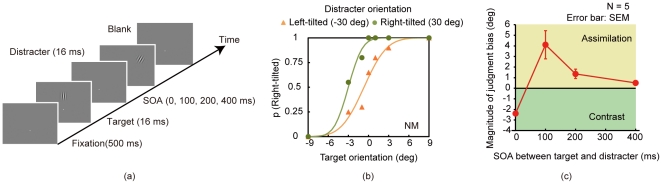
Experiment 1. (a) Schematic diagram of an experimental trial in Experiment 1. (b) Representative data in the 100-ms-SOA condition. (c) Group mean of the magnitude of judgment bias (N = 5). Error bars denote standard errors of mean.

The purpose of this experiment was to explore whether the judgment of visual orientation was postdictively modulated. The observers were sequentially presented two tilted Gabor patches: the first patch as a target and the second one as a distracter (See Methods section and Figure 1a for details). The task of the observers was to judge whether the target was right- or left-tilted. We controlled stimulus onset asynchronies between the target and distracter in four levels (0, 100, 200, and 400 ms). We controlled the SOA between the target and distracter because previous studies have demonstrated that postdictive modulation of visual appearance is based on the spatial integration of visual signals within ∼100 ms [3], [10], [23], [24]. We expected that the postdictive modulation of the visual orientation appearance would peak at the 100-ms SOA between the target and distracter.

The proportion of trials in which the observers reported the right-tilted target was calculated for each target orientation. The proportions of a representative observer are shown in Figure 1b. A cumulative Gaussian function was fitted to the proportion as a function of target orientation. We calculated points of subjective equality (PSE) of the target orientation for each distracter orientation condition and each target-distracter SOA condition. We subtracted the PSE in the right-tilted distracter condition from the one in the left-tilted distracter condition, and calculated the magnitude of judgment bias (Figure 1c). The positive and negative values of the magnitude represent the orientation judgment that is attractive toward and repulsive from the distracter, respectively. The attractive orientation judgment bias indicates the postdictive modulation of visual orientation.

The group mean of the magnitude of judgment bias was submitted to a one-way ANOVA with the SOA between the target and distracter as a factor. The main effect was significant [*F*(3, 12) = 11.306, *p*<.05]. Multiple comparison tests (Ryan's method [31]) showed that the magnitude of judgment bias in the 100-ms-SOA condition was significantly larger than that in the 0- and 400-ms-SOA conditions (*p*<.05), and was marginally larger than that in the 200-ms-SOA condition (*p*<.1). The magnitude of judgment bias in the 0-ms SOA-condition was significantly smaller than that in the 200- and 400-ms-SOA conditions (*p*<.05). A one-sample t-test showed that the magnitude of judgment bias was significantly larger than zero in the 100- and 200-ms-SOA conditions [*t*s(5) = 2.83 and 2.83, respectively, *p*<.04] and significantly smaller than zero in the 0-ms-SOA condition [*t*(5) = 4.50, *p*<.007].

The results suggest that the judgment of visual orientation can be modulated by subsequent orientation inputs. Moreover, the magnitude of the judgment bias peaked at 100 ms. The SOA with the peak of the postdictive modulation was consistent with that for previously reported postdictive modulation [3], [10], [23], [24].

Interestingly, the judgment bias was significantly reversed in the 0-ms-SOA condition. We suggest that a simultaneous orientation contrast occurred in this condition. A simultaneous orientation contrast refers to a perceptual phenomenon that a cardinal orientation in a central area is perceptually repulsed from the tilted surround [32]. In the 0-m-SOA condition, the target and distracter appeared and disappeared at the same time, sufficing a stimulus condition for the simultaneous motion contrast [33].

In the 400-ms SOA condition, the magnitude of orientation judgments did not significantly deviate from zero. The results indicate that the observers were not simply inclined to report the orientation of the distracter, which is consistent with the previous finding demonstrating that postdictive modulation of visual size does not stem from the response bias toward the subsequent information [11]. Thus, we suggest that temporal integration of visual orientation on the basis of feature matching perhaps underlie the postdictive modulation of visual orientation.

However, it was still possible that the peak of the postdictive modulation at the100-ms-SOA condition might have stemmed from response bias. It is well known that the strength of backward masking peaks at approximately 100 ms SOA between a target and a masker [16]. Thus, one may argue that because the target visibility might have been weakened maximally at the 100 ms SOA, and hence the uncertainty of the target orientation was highest, leading to the largest response bias towards the distracter orientation. We addressed this issue in the next experiment.

### Supplementary experiment

The purpose of this experiment was to exclude the possibility that the postdictive modulation of visual orientation stemmed from response bias for the observers to report distracter orientation as target orientation. Following a previous study [11], we employed a verticality judgment task in which the observers were asked to report whether the target was vertical or not. In this task, the observer would not simply rely their responses on the distracter orientation because the observers did not directly judge the tilt direction of the target. If the postdictive modulation stemmed from temporal integration of target and distracter orientation, the function of verticality judgment for the target would have a peak at a specific target orientation, and the peak would shift in the opposite tilt direction of the distracter. On the other hand, if the observers relied their response for target orientation solely on distracter orientation, they would have a strong bias to report that the target was not vertical because the distracter always titled ±30 deg while no shift of the peak of the function would be observed.

**Figure 2:**
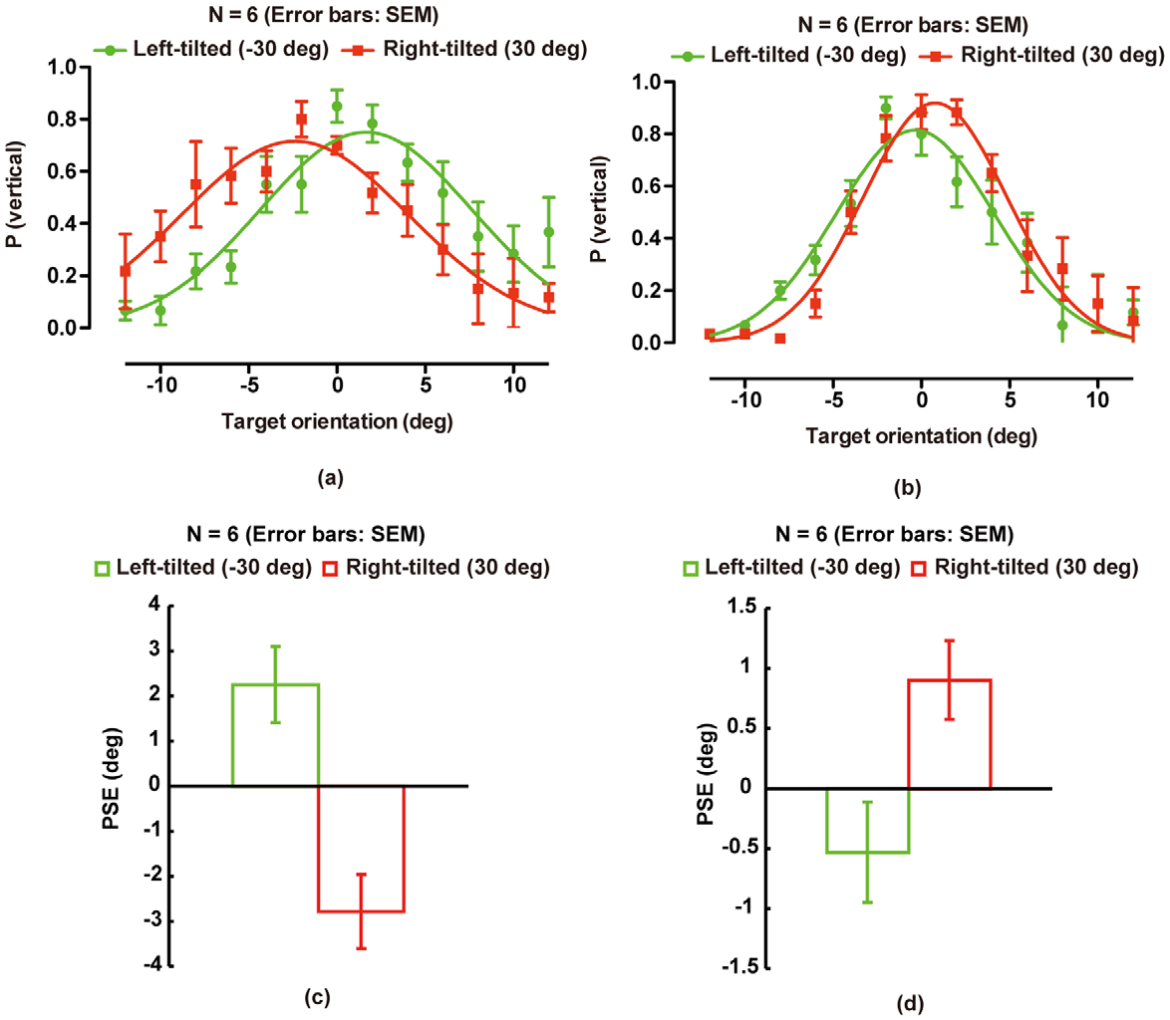
Supplementary experiment. (a, b) Group mean of the proportions of trials in which the target was reported to be vertical in (a) the 100- and (b) 0-ms-SOA conditions as a function of target orientation. (c, d) Group mean of the magnitude of PSEs for each distracter orientation (N = 7) in (a) the 100- and (b) 0-ms-SOA conditions, respectively. Error bars denote standard errors of mean.

The proportion of trials in which the observers reported the target to be vertical was calculated for each target orientation. Group data of the proportion are shown in Figures 2a and 2b for the 100- and 0-ms-SOA conditions, respectively. A Gaussian function was fitted to the proportion as a function of target orientation, and we calculated the peak location of the function as PSE of the target orientation for each distracter orientation and each target-distracter SOA condition (Figures 2c and 2d for PSE in the 100- and 0-ms-SOA conditions, respectively). As a result of a two-tailed paired t-test, in the 100-ms-SOA condition, the PSE was significantly different between the distracter orientation conditions [*t*(5) = 3.06, *p*<.03]. Moreover, in the 0-ms-SOA condition, the PSE was significantly different between the distracter orientation conditions [*t*(5) = 5.02, *p*<.005].

The results are well consistent with those obtained in Experiment 1. In the 100-ms-SOA condition, PSE shifted in the direction opposite to distracter orientation, suggesting that the judgment of target orientation was postdictively modulated by distracter orientation even in a bias-free task. In the 0-ms-SOA condition, PSE shifted in the direction similar to distracter orientation, indicating that the simultaneous orientation contrast occurred as in Experiment 1. Thus, we suggest that the postdictive modulation (as well as the simultaneous orientation contrast) occurs even when the involvement of response bias is excluded. On the other hand, we noticed that as indicated by relatively large error bars in Figure 2a, the verticality judgment task was more susceptive to an arbitrary criterion for the verticality in each observer than a usual 2AFC orientation judgment task as used in Experiment 1. Thus, we suspected that the verticality judgment task could not have detected a subtle change in the appearance of visual orientation due to an arbitrary criterion in each observer. For these reasons, we again employed the 2AFC task in the following experiment.

### Experiment 2

The purpose of this experiment was to explore how the visibility of the distracter altered the magnitude of the postdictive modulation of visual orientation. We hypothesized that the postdictive modulation occurs on the basis of motion correspondence determined by higher-order feature matching across space and time [25], [34]. Hence, we predicted that the postdictive modulation would be reduced in a low visibility distracter condition if it depended on higher-order feature matching. For a control condition, we also examined whether the magnitude of the simultaneous orientation contrast was influenced by the visibility of the distracter. As described in the introduction, visual orientation is processed without awareness of it in the brain [26]–[30], and unconscious orientation signals contribute to the simultaneous orientation contrast [26], [29]. Thus, the control condition served as a good index to confirm whether the low visibility distracter had enough effective strength to stimulate orientation detectors.

In the first experiment phase, we first asked the observers to judge the tilt of the distracter that was subsequently masked by a plaid pattern (a target was not presented). SOAs between the distracter and the plaid masker were controlled in seven levels. We calculated the proportion of trials in which the observer could correctly report the distracter tilt, and fitted a cumulative Gaussian curve to the proportion as a function of SOA. For each individual, we calculated the SOA producing 60% and 90% correct responses. The group mean of the SOA producing 60% correct responses was 35.72 ms (SD: 13.35 ms) and the one producing 90% correct responses was 109.6 ms (SD: 29.11 ms). They were significantly different from each other [*t*(6) = 5.706, *p*<.001].

**Figure 3:**
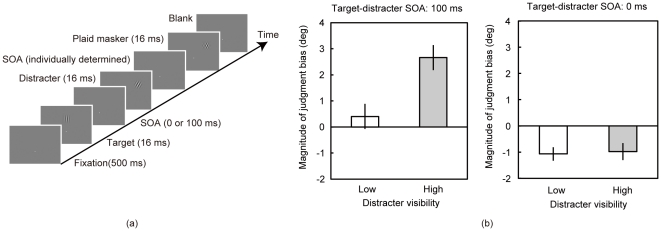
Experiment 2. (a) Schematic diagram of an experimental trial in Experiment 2. (b) Group mean of the magnitude of judgment bias (N = 7). Left and right panels show data in the 100- and 0-ms-SOA conditions, respectively. Error bars denote standard errors of mean.

By using the SOAs, in the second experimental phase, we assessed the role of visibility in the postdictive modulation of visual orientation. The observers were presented the target, the distracter, and the plaid masker, and asked to report whether the target was right- or left tilted (Figure 3a). In the low visibility condition, the SOA between the distracter and the plaid masker was individually set to the one producing 60% correct responses for the distracter tilt. In the high visibility condition, the SOA was individually set to the one producing 90% correct responses. Two SOAs (0 and 100 ms) between the target and distracters were employed.

The proportion of trials in which the observers reported the target to be the right-titled was calculated for each target orientation, and as in Experiment 1, we calculated the PSE difference between the right- and left-tilted distracter conditions as the magnitude of judgment bias (Figure 3b). In the 100-ms-SOA condition, there was a significant difference in the magnitude of judgment bias between the low and high visibility conditions [*t*(6) = 4.49, *p*<.005]. In addition, a one-sample t-test showed that the judgment bias was significantly biased toward the distracter orientation in the high- visibility condition [*t*(6) = 4.46, *p*<.005] but not in the low-visibility condition [*t*(6) = 1.00, *p*>.35]. In the 0-ms-SOA condition, there was no significant difference in the magnitude of judgment bias between the low- and high-visibility conditions [*t*(6) = 0.21, *p*>.8]. A one-sample t-test showed that the magnitude of judgment bias was significantly different from the distracter orientation in both the high- and low-visibility conditions [*t*(6) = 3.23, *p*<.02 and *t*(6) = 4.85, *p*<.003, respectively]

The results suggest that the postdictive modulation of visual orientation is dependent on the visibility of subsequent orientation signals. The results are in accord with a previous study showing that motion correspondence, which likely underlies the postdictive modulation of visual appearance, is determined by a slow feature matching process [34]. In the high-visibility condition, the magnitude of judgment bias was significantly higher than zero, successfully replicating the results of Experiment 1. In contrast, consistent with some previous studies [26], [29], the simultaneous orientation contrast occurred even when the visibility of the distracter was low. The results indicate that the orientation signals was strong enough to stimulate the mechanism responsible for spatial integration of visual orientation but not sufficient for driving the postdictive modulation of visual orientation appearance. The results indicate that a neural site for the postdictive modulation of visual appearance is located beyond the neural site for the spatial orientation integration.

## Discussion

The present study investigated when and how the postdictive modulation of visual orientation occurs. The results of Experiment 1 demonstrated that the postdictive modulation of visual orientation did occur and that it peaked at the 100 ms SOA between the target and distracter. The results of Experiment 2 showed that the postdictive modulation of visual orientation vanished, while the simultaneous orientation contrast still occurred, when backward masking lowered the visibility of the distracter. The results indicate that the postdictive modulation of visual orientation stems from object-updating causing the spatiotemporal integration of orientation signals within ∼100 ms.

Several previous studies have also suggested the role of feature matching in the object updating leading to the postdictive modulation of visual appearance. It has been suggested that the spatiotemporal integration of visual signals within ∼100 ms is one of causes for the flash-lag effect, where a transient flash with a spatial alignment to a moving object is perceived to spatially lag behind the moving object [4], [35], [36]. However, when a visual feature of a moving object is changed immediately after the flash, the flash-lag effect vanishes [37]. Given the visual feature change, the visual system quits updating the moving object, leading to the maintenance of visible persistence along a motion trajectory [17], [37]. In other words, the visual system continuously monitors the continuity of visual features across space and time, and the destruction of the continuity of visual features across space and time also hampers object-updating. In the present study, the postdictive modulation of visual orientation vanished when the visibility of the distracter was lowered. The results of the present study indicate that the feature matching required for object updating deteriorated due to the lowered visibility of the distracter, and thus the postdictive modulation did not occur. In a similar vein, the necessity for the visual awareness of the surround information in the postdictive modulation has been reported in a study on motion-induced position shift [38].

How was the disappearance of the postdictive modulation in the low-distracter-visibility condition related to the target recovery effect reported in backward masking studies (e.g., [39]–[41])? The target recovery effect is a phenomenon where the reportability of a masked target increases when a task-irrelevant flash is presented immediately after the mask. In our Experiment 2, the distracter serving as the metacontrast masker may have been masked by the plaid masker, and this might have restored the visibility of the target, inhibiting the postdictive modulation. Although the target recovery effect possibly occurred in our stimuli, it might not be a source of the disappearance of the postdictive modulation. We suggest that the reason why the target recovery effect occurred in our stimuli may be because the object formation between the target and distracter was hampered due to the masking of the distracter by the plaid masker and thus the object updating was simultaneously hampered. Moreover, the reason why the object formation between the target and distracter was deteriorated might be because neural signal strength of the distracter was weakened via the plaid masker, and hence the visual system could not match visual features between the target and distracter. Taken together, it is likely that the disappearance of the postdictive modulation in Experiment 2 stemmed from the deterioration of the object formation between the target and distracter due to the plaid masker, which diminished the object updating underlying the postdictive modulation. In this way, the target recovery effect is not a cause of the disappearance of the postdictive modulation, but a result of the deterioration of the object updating.

The low visibility distracter did not cause the postdictive modulation of visual orientation, despite that it caused the simultaneous motion contrast. As described above, feature matching across space and time is mediated by a slow comparison process [25], [42]–[44]. We suggest that the slow feature matching depends on the reentrant (or recurrent) information processing in the brain. It has been discussed that a reentrant visual process underlies object updating [45]. Moreover, backward masking interrupts recurrent processing in the brain (e.g. [46], [47]). Thus, it is possible that the plaid masker disrupted the recurrent processing required for feature matching and object updating, leading to the absence of the postdictive modulation of visual orientation. Since it has been argued that the recurrent processing is required for the occurrence of visual awareness [48], it is tentatively hypothesized that the postdictive modulation of visual appearance requires visual awareness for subsequent input signals. Future studies should attempt to specify the precise relationship among the postdictive modulation, object-updating, and visual awareness.

## Materials and Methods

### Ethics Statement

Ethical approval for this study was obtained from the ethical committee at Nippon Telegraph and Telephone Corporation (NTT Communication Science Laboratories Ethical Committee). The experiments were conducted according to the principles laid down in the Helsinki Declaration. Written informed consent was obtained from all participants except the author.

### Experiment 1

#### Participants

Five people including the author participated in this experiment. They reported they had normal or corrected-to-normal visual acuity.

#### Apparatus

Stimuli were presented on a 21-inchi CRT monitor (GDM-F500R, Sony) with the resolution of 1024×768 pixels (38×30 cm) and the refresh rate of 60 Hz. A photometer (OP200-E, Cambridge Research Systems) linearlized the luminance emitted from the monitor in a range from 0 to 106 cd/m^2^. A computer (Mac pro, Apple) controlled stimulus generation, stimulus presentation, and data collection. Stimuli were generated by using MATLAB and PsychToolBox 3 [49], [50]. The observers used a chin-head rest to stabilize their visual field.

#### Stimuli

Stimuli consisted of a gray background (53 cd/m^2^), a fixation dot (a 0.15×0.15 deg green dot), and a Gabor patch. For each patch, a luminance sinusoidal wave with the spatial frequency of 1.82 cycles per degree was Gaussian-windowed with a standard deviation of 0.72 deg. The phase and Michelson contrast of the sinusoidal wave were 0.5 π and 0.8, respectively. The target Gabor patch (i.e., the target) was presented 3.55 deg above the fixation dot. The distracter Gabor patch (i.e., the distracter) was presented 3.55 deg left or right of the target. The spatial side of the distracter was also randomized across trials. Stimulus onset asynchrony (SOA) between the target and distracter was randomly chosen from 0 (simultaneous), 100, 200, or 400 ms. The orientation of the target was randomly selected from −9, −3, −1, 0, 1, 3, or 9°, where negative and positive values represent left- and right-tilted orientation, respectively. The orientation of the distracter was −30 or 30°, which was randomly chosen from trial to trial.

#### Procedure

The experiment was conducted in a dark room. The observer sat at the 62 cm from the CRT monitor. An experimental session started when the observer pressed the spacebar on the keyboard of the computer. Intervening a 500-ms blank period with the fixation dot against the background, the target was presented for one frame (16.7 ms). Subsequently, with the SOA (0, 100, 200, or 400 ms), the distracter was presented for one frame (16.7 ms). The task of the observer was to judge whether the target was right- or left-tilted by pressing assigned keys. The observers were allowed to report only after the distracter disappeared (i.e., the key was not active unless the distracter disappeared). They were also urged to maintain their gaze on the central fixation dot while the stimuli were presented (the fixation dot was presented all through the block). Each observer performed 1,120 trials consisting of 4 SOAs×2 distracter orientations (−30 or 30°)×7 target orientations×20 repetitions. Each SOA condition was blocked. The order of blocks was randomized across observers. Moreover, the order of trials was also randomized across blocks as well as observers. A short break (∼15 minutes) was inserted between blocks. Thus, it took two hours for each observer to complete this experiment.

### Supplementary experiment

#### Participants

Six people including the author participated in this experiment. They reported they had normal or corrected-to-normal visual acuity.

#### Apparatus

The apparatus was identical to that used in Experiment 1.

#### Stimuli

The stimuli were identical to those used in Experiment 1 except for the following. Two SOAs (0 and 100 ms) between the target and distracter were employed because we wanted to clarify whether the postdictive modulation occurred even when the response bias was expected to be strongest (i.e. at the 100 ms SOA), and because we liked to know whether simultaneous orientation contrast was also obtained in a bias-free paradigm (i.e. at the 0 ms SOA). The orientation of the target was randomly selected from −12, −10, −8, −6, −4, −2, 0, 2, 4, 6, 8, 10, and 12°, where negative and positive values represent left- and right-tilted orientation, respectively. As a reference of verticality, another vertical grating with identical parameter to the target was provided 3.55 deg below the fixation point. The reference was presented from the initiation of each trial to the offset of the distracter.

#### Procedure

The procedure was identical to that used in Experiment 1 except for the following. The task of the observer was to report whether the target was vertical or not. Each observer performed 520 trials of 2 (the distracter orientation)×2 SOAs×13 target orientations×10 repetitions. The trial order was randomized across observers. It took a half hour for each observer to complete this experiment.

### Experiment 2

#### Participants

Seven people including the author participated in this experiment. They reported they had normal or corrected-to-normal visual acuity.

#### Apparatus

The apparatus was identical to that used in Experiment 1.

#### Stimuli

Basically, the stimuli were identical to those used in Experiment 1 except for the following. Two SOAs (0 and 100 ms) between the target and distracter were employed because they produced the strongest orientation contrast as well as the strongest postdictive orientation modulation in Experiment 1. A plaid masker was additionally employed. The plaid masker consisted of the summation (in a contrast dimension) of two sinusoidal gratings tilted −30 and 30°. The ratio of the contrast of each grating was 0.5∶0.5. The spatial frequency and phase of each grating were 1.82 cpd and 0.5π, respectively. The plaid was Gaussian-windowed with the standard deviation of 0.72 deg. The plaid masker was presented at the distracter location after the distracter disappeared. SOAs between the distracter and the plaid masker were individually determined (See details in Procedure). The duration of the plaid masker was 30 frames (500 ms)

#### Procedure

The procedure was identical to that used in Experiment 1 except for the following. The experiment had two experimental phases. In the first phase, the performance for the distracter orientation discrimination was assessed for different SOAs between the distracter and the plaid masker. Seven SOA levels were tested (17, 33, 66.6, 100, 133, 200, and 400 ms). The task of the observers was to report whether the distracter was right- or left-tilted. A cumulative Gaussian curve was individually fitted to the correct proportion of orientation judgments as a function of SOA, and the SOAs producing 60% and 90% correct orientation judgments were calculated. Each observer performed 560 trials of 2 (the distracter orientation)×7 SOAs×40 repetitions. The trial order was randomized across observers. In the second phase, as in Experiment 1, the observers were asked to report whether the target was right- or left tilted. In this experiment, however, the plaid masker was presented at the distracter location with the SOAs that were calculated in the first experimental phase. Each observer performed 1120 trials of 2 (the SOA between the target and distracter)×2 (the SOA between the distracter and the plaid masker)×2 (the distracter orientation)×7 target orientations×20 repetitions. It took two and a half hours for each observer to complete this experiment.

## References

[1] Fu Y-X, Shen Y, Dan Y (2001). Motion-induced perceptual extrapolation of blurred visual targets.. J Neurosci.

[2] Kerzel D (2003). Attention maintains mental extrapolation of target position: Irrelevant distractors eliminate forward displacement after implied motion.. Cognition.

[3] Eagleman DM, Sejnowski TJ (2000). Motion integration and postdiction in visual awareness.. Science.

[4] Fröhlich FW (1923). Über die messung der empfindungszeit.. Zeitschrift für Sinnesphysiologie.

[5] Kolers PA, Pomerantz JR (1971). Figural changes in apparent motion.. J Exp Psychol.

[6] Kolers PA, von Grunau M (1975). Visual construction of color is digital.. Science.

[7] Kolers PA (1984). Motion from continuous or discontinuous arrangements.. ACM SIGGRAPH Computer Graphics.

[8] Kolers PA, von Grunau M (1976). Shape and color in apparent motion.. Vison Res.

[9] Nishida S, Watanabe J, Kuriki I, Tokimoto T (2007). Human brain integrates color signals along motion trajectory.. Curr Biol.

[10] Eagleman DM, Sejnowski TJ (2007). Motion signals bias position judgments: A unified explanation for the flash-lag, flash-drag, flash-jump and Fröhlich effects.. J Vision.

[11] Kawabe T (2011). Nonretinotopic processing is related to postdictive size modulation in apparent motion.. Atten Percept Psycho.

[12] Carlson TA, Rauschenberger R, Verstraten FAJ (2007). No representation without awareness in the lateral occipital cortex.. Psychol Sci.

[13] Di Lollo V, Enns JT, Rensink RA (2000). Competition for consciousness among visual events: The psychophysics of reentrant visual processes.. J Exp Psychol Gen.

[14] Enns JT, Di Lollo V (1997). Object substitution: A new form of masking in unattended visual locations.. Psychol Sci.

[15] Lleras A, Moore CM (2003). When the target becomes the mask: Using apparent motion to isolate the object-Level component of object substitution masking.. J Exp Psychol: Human.

[16] Enns JT (2004). Object substitution and its relation to other forms of visual masking.. Vision Res.

[17] Moore CM, Mordkoff JT, Enns JT (2007). The path of least persistence: Object status mediates visual updating.. Vision Res.

[18] Hirose N, Kihara K, Mima T, Ueki Y, Fukuyama H (2007). Recovery from object substitution masking induced by transient suppression of visual motion processing: A repetitive transcranial magnetic stimulation study.. J Exp Psychol Human.

[19] Burr DC (1980). Motion smear.. Nature.

[20] Burr DC (1984). Summation of target and mask metacontrast stimuli.. Perception.

[21] Burr DC, Ross J, Morrone MC (1986). Seeing objects in motion.. P Roy Soc Lond B Bio.

[22] Kahan TA, Enns JT (2010). Object trimming: When masking dots alter rather than replace target representation.. J Exp Psychol Human.

[23] Arnold DH, Thompson M, Johnston A (2007). Motion and position coding.. Vision Res.

[24] Chung STL, Patel SS, Bedell HE, Yilmaz O (2007). Spatial and temporal properties of the illusory motion-induced position shift for drifting stimuli.. Vision Res.

[25] Kawachi Y, Kawabe T, Gyoba J (2011). Stream/bounce event perception reveals a temporal limit of motion correspondence based on surface feature over space and time.. i-Perception.

[26] Clifford CWG, Harris JA (2005). Contextual modulation outside of awareness.. Curr Biol.

[27] He S, MacLeod DI (2001). Orientation-selective adaptation and tilt after-effect from invisible patterns.. Nature.

[28] Kanai R, Tsuchiya N, Verstraten FAJ (2006). The scope and limits of top-down attention in unconscious visual processing.. Curr Biol.

[29] Motoyoshi I, Hayawaka S (2010). Adaptation-induced blindness to sluggish stimuli.. J Vis.

[30] Rajimehr R (2004). Unconscious orientation processing.. Neuron.

[31] Ryan TA (1960). Significance tests for multiple comparison of proportions, variances, and other statistics.. Psychol Bull.

[32] Gibson JJ, Radner M (1937). Adaptation, after-effect, and contrast in the perception of tilted lines. I. Quantitative studies.. J Exp Psychol.

[33] Corbett JE, Handy TC, Enns JT (2009). When do we know which way is up? The time course of orientation perception.. Vision Res.

[34] Prins N (2008). Correspondence matching in long-range apparent motion.. Perception.

[35] Nijhawan R (1994). Motion extrapolation in catching.. Nature.

[36] MacKay D (1958). Perceptual stability of a stroboscopically lit visual field containing self-luminous objects.. Nature.

[37] Moore CM, Enns JT (2004). Object updating and the flash-lag effect.. Psychol Sci.

[38] Watanabe K (2005). The motion-induced position shift depends on the visual awareness of motion.. Vision Res.

[39] Robinson DN (1966). Disinhibition of visually masked stimuli.. Science.

[40] Tenkink E, Werner JH (1981). The intervals at which homogeneous flashes recover masked targets.. Percept Psychophys.

[41] Öğmen H, Breitmeyer BG, Todd S, Mardon L (2006). Target recovery in metacontrast: The effect of contrast.. Vision Res.

[42] Enns JT (2002). Visual binding in the standing wave illusion.. Psychon Bull Rev.

[43] Holcombe AO (2009). Seeing slow and seeing fast: two limits on perception.. Trends Cogn Sci.

[44] Holcombe A, Cavanagh P (2001). Early binding of feature pairs for visual perception.. Nature Neurosci.

[45] Di Lollo V, Enns JT, Rensink RA (2000). Competition for consciousness among visual events: the psychophysics of reentrant visual processes.. J Exp Psychol Gen.

[46] Fahrenfort JJ, Scholte HS, Lamme VAF (2007). Masking disrupts reentrant processing in human visual cortex.. Journal Cognitive Neurosci.

[47] Boehler CN, Schoenfeld MA, Heinze HJ, Hopf JM (2008). Rapid recurrent processing gates awareness in primary visual cortex.. P Natl Acad Sci USA.

[48] Lamme VAF (2011). How neuroscience will change our view on consciousness.. Cogn Neurosci.

[49] Brainard DH (1997). The Psychophysics Toolbox.. Spatial Vision.

[50] Pelli DG (1997). The VideoToolbox software for visual psychophysics: Transforming numbers into movies.. Spatial Vision.

